# Longitudinal association between physical activity and health-related quality of life among community-dwelling older adults: a longitudinal study of Urban Health Centres Europe (UHCE)

**DOI:** 10.1186/s12877-021-02452-y

**Published:** 2021-10-01

**Authors:** Xuxi Zhang, Siok Swan Tan, Carmen Betsy Franse, Tamara Alhambra-Borrás, Arpana Verma, Greg Williams, Amy van Grieken, Hein Raat

**Affiliations:** 1 Department of Public Health, Erasmus University Medical Center, P.O. Box 2040, 3000 CA Rotterdam, The Netherlands; 2 Center for Healthy Aging and Development Studies, National School of Development, Peking University, Beijing, 100871 China; 3 Polibienestar Research Institute, Universitat de València, 29 46022 Valencia, Spain; 4 Manchester Urban Collaboration on Health, Centre for Epidemiology, Division of Population Health, Health Services Research and Primary Care, Manchester Academic Health Science Centre, The University of Manchester, Manchester, UK

**Keywords:** Physical activity (PA), Longitudinal study, Health-related quality of life (HRQoL), Physical HRQoL, Mental HRQoL, Older adults

## Abstract

**Background:**

Physical activity (PA) may play a key role in healthy aging and thus in promoting health-related quality of life (HRQoL). However, longitudinal studies on the association between PA and HRQoL are still scarce and have shown inconsistent results. In this study, we aimed to examine the longitudinal association between frequency of moderate PA and physical and mental HRQoL. Secondly, to assess the association between a 12-month change in frequency of moderate PA and HRQoL.

**Methods:**

A 12-month longitudinal study was conducted in Spain, Greece, Croatia, the Netherlands, and the United Kingdom with 1614 participants (61.0% female; mean age = 79.8; SD = 5.2) included in the analyses. Two categories of the self-reported frequency of moderate PA including 1) ‘regular frequency’ and 2) ‘low frequency’ were classified, and four categories of the change in frequency of moderate PA between baseline and follow-up including 1) ‘continued regular frequency’, 2) ‘decreased frequency’, 3) ‘continued low frequency’ and 4) ‘increased frequency’ were identified. Physical and mental HRQoL were assessed by the 12-Item Short-Form Health Survey (SF-12).

**Results:**

The frequency of moderate PA at baseline was positively associated with HRQoL at follow-up. Participants with a continued regular frequency had the highest HRQoL at baseline and follow-up. Participants who increased the frequency of moderate PA from low to regular had better physical and mental HRQoL at follow-up than themselves at baseline. After controlling for baseline HRQoL and covariates, compared with participants who continued a regular frequency, participants who decreased their frequency had significantly lower physical (B = -4.42; *P < .001*) and mental (B = -3.95; *P < .001*) HRQoL at follow-up; participants who continued a low frequency also had significantly lower physical (B = -5.45; *P < .001*) and mental (B = -4.10; *P < .001*) HRQoL at follow-up. The follow-up HRQoL of participants who increased their frequency was similar to those who continued a regular frequency.

**Conclusions:**

Maintaining or increasing to a regular frequency of PA are associated with maintaining or improving physical and mental HRQoL. Our findings support the development of health promotion and long-term care strategies to encourage older adults to maintain a regular frequency of PA to promote their HRQoL.

**Supplementary Information:**

The online version contains supplementary material available at 10.1186/s12877-021-02452-y.

## Background

Health-related quality of life (HRQoL) is a multidimensional construct that specifically focuses on health-related aspects of well-being and reflects the subjective perception of the impact of physical and mental functioning on a person’s daily living [1]. HRQoL is an important component in determining the health status of older adults during the aging process [2]. Population aging is associated with increased morbidity and institutionalization, which may adversely affect the HRQoL among older adults [3]. Physical activity (PA) may play a key role in healthy aging and thus in promoting HRQoL [2]. A better understanding of the association between PA and HRQoL among older adults might help policy makers to develop more precise policies for long-term care and healthy aging [4].

Previous cross-sectional studies have shown consistent positive associations between PA and HRQoL [5, 6]. However, longitudinal studies on the association between PA and HRQoL are still scarce and have shown inconsistent results [6, 7]. Some longitudinal studies reported that PA was positively associated with better physical and mental HRQoL [8, 9]. Conversely, some studies reported PA was not associated with improved physical or mental HRQoL [10, 11]. In addition, few studies focused on the longitudinal association between change in PA and HRQoL, [12] which is an important scientific evidence for the development of health promotion and long-term care strategies. Furthermore, studies on the association between PA and HRQoL among older adults, especially people aged over 70, are relatively limited [2]. More studies are needed to have a better understanding of the longitudinal association between PA and HRQoL in older populations.

Therefore, the aim of our study is to examine the longitudinal association between frequency of moderate PA and physical and mental HRQoL among community-dwelling older adults aged over 70. Secondly, we assessed the association between a 12-month change in frequency of moderate PA and HRQoL.

## Methods

### Participants

The Urban Health Centres Europe (UHCE) project aimed to promote the healthy aging of older adults using integrated care pathways in community settings at study sites in five European countries, including Spain, Greece, Croatia, the Netherlands, and the United Kingdom [13–15]. At each study site, older adults over age 70, who lived independently and were expected to be able to participate in the study for at least 6 months were invited to participate. A total of 2325 participants were recruited between May 2015 and June 2017, of which 1215 received integrated care pathways (intervention) and 1110 were enrolled in the control group. At the 12-month follow-up, 986 in the intervention group (81.2%) and 858 in the control group (77.3%) completed the questionnaire [13]. Participants in the intervention group received care following the UHCE approach which comprised of three elements: 1) risk assessment, 2) shared-decision making and 3) referral to care pathways aimed at reducing fall risk, inappropriate medication use, loneliness or frailty by specific interventions [13]. Persons in the control group received their usual care [13]. Further details on these interventions are described elsewhere [13, 14]. Data were obtained from self-reported questionnaires at baseline and 12 months of follow-up. Ethics committee procedures were followed at all study sites and approval was provided [13, 14]. Written informed consent was obtained from all participants [13]. The study was registered in the ISRCTN registry as ISRCTN52788952.

The current study included participants in the UHCE project who had completed both baseline and follow-up questionnaires (*n* = 1844) [13]. We excluded participants with missing data on PA (*n* = 71) or HRQoL (*n* = 121) or Age (n = 1). Additionally, participants whose age < 70 years (*n* = 37) were excluded. Thus, a total of 1614 participants were included in the analyses of the current study.

Compared with the study population (*n* = 1614), the participants excluded from the study (*n* = 230) were younger (*P < .001*) and had less often completed secondary or higher education (*P < .001*; details on how to define ‘secondary or higher education’ can be found in the following section). No significant differences in sex, living situation, smoking, alcohol risk, frailty, and multi-morbidity between these two groups were found.

### Measurements

#### Physical activity (PA)

The frequency of moderate PA was measured using one question from the Frailty Instrument of the Survey of Health, Aging and Retirement in Europe (SHARE-FI): “How often do you engage in activities that require a low or moderate level of energy such as gardening, cleaning the car, or taking a walk?”. Answer categories included (a) more than once a week, (b) once a week, (c) one to three times a month and (d) hardly ever, or never. If the answer falls into the frequency of “more than once a week”, it would be considered as ‘Regular frequency’ (more than once a week). If the answer was “once a week”, “one to three times a month”, or “hardly ever, or never”, it would be taken as ‘Low frequency’ (once a week or less). In addition, the change in the frequency between baseline and follow-up was grouped into four categories: (1) ‘Continued regular frequency’ (more than once a week), (2) ‘Decreased frequency’ (from regular to low frequency), (3) ‘Continued low frequency’ (once a week or less) and (4) ‘Increased frequency’ (from low to regular frequency) [16].

#### Health-related quality of life (HRQoL)

Physical and mental HRQoL were measured by the SF-12. It is a widely used patient-reported survey for measuring HRQoL which consists of 12 questions covering eight health domains, including general health, mental health, vitality, social functioning, role limitation due to physical health problems, role limitation due to emotional problems, bodily pain limiting usual activities, and physical functioning [17]. The eight domains of SF-12 can be summarized in the Physical Component Summary (PCS; physical HRQoL) and Mental Component Summary (MCS; mental HRQoL), both ranging from 0 (lowest) to 100 (highest level of health) [17, 18]. A 3-unit change or more in PCS and MCS are considered clinically important changes [19].

#### Covariates

We assessed covariates including age (in years), sex, country, educational level, living situation, smoking, alcohol risk, frailty, and multi-morbidity at baseline. Educational level concerned the highest level of education completed by the participant. It was categorized according to the 2011 International Standard Classification of Education (ISCED) into primary or less (ISCED 0–1), secondary or equivalent (ISCED 2–5), and tertiary or higher (ISCED 6–8) [20]. The living situation was categorized as ‘not living with others’ or ‘living with others’ (a partner, child (ren) and/or others). Smoking was measured with one item that assessed whether a person currently smoked. Alcohol risk was measured with the AUDIT-C, [21] which is a 3-item screener to grade high-risk alcohol use on a scale from 0 (lowest risk) to 12 (highest risk). A score of at least 4 for men and 3 for women was regarded as hazardous drinking or active alcohol use disorder [21]. Frailty was measured with the Tilburg Frailty Indicator (TFI), which is a reliable and validated instrument to identify frailty in community-dwelling older adults [22]. An overall frailty score can be determined by adding up the 15 items (score range 0–15), and participants with a total score of at least five were diagnosed as being frail [23]. Multi-morbidity was defined as having at least two of the following 14 chronic conditions [24]: heart attack, hypertension, diabetes, stroke, high blood cholesterol, asthma, arthritis, osteoporosis, chronic lung disease, cancer or malignant tumor, stomach or duodenal ulcer, Parkinson’s disease, cataract and hip fracture or femoral fracture [25].

### Statistical analysis

To examine mean differences in PCS and MCS scores between groups, effect sizes were estimated by calculating the mean difference between two subgroups, and then dividing the result by the pooled standard deviation. Cohen’s effect sizes (d) were used for the interpretation of relevant differences: 0.20 ≤ d < 0.50 was considered a small difference; 0.50 ≤ d < 0.80 was considered a moderate difference; d ≥ 0.80 was considered a large difference [26].

The longitudinal association between frequency of moderate PA and HRQoL was estimated with multivariate linear regression models. Two separate regression models were built for physical and mental HRQoL at 12 months of follow-up as the dependent variable, and frequency of moderate PA at baseline as the independent variable. The first set of models were adjusted for HRQoL at baseline and country (crude model). The second set of models were additionally adjusted for age, sex, educational level, living situation, smoking, alcohol risk, frailty, and multi-morbidity (adjusted model) [16]. Since the UHCE project was an intervention study, and participants were divided over an intervention and a control group, intervention condition (yes/no) was also added to the adjusted model as a covariate [16].

The association between the 12-month change in frequency of moderate PA and physical or mental HRQoL was assessed using the same crude and adjusted multivariate linear regression models as described above, taking the change in frequency of moderate PA as the independent variable [16].

Furthermore, interactions between baseline frequency of moderate PA or 12-month change in frequency of moderate PA and age, sex, country, educational level, living situation and intervention condition (yes/no) on HRQoL were assessed with UNIANOVA [16]. Bonferroni correction was applied for multiple testing (*P* = 0.05/24 = 0.002). Apart from an interaction between change in frequency of moderate PA and country regarding mental HRQoL, no statistically significant interaction was found. All *P*-values of the interaction analyses are presented in Additional file 1: Table A1.

Finally, sensitivity analyses were performed where all analyses were repeated using the participants in the control group only; we found comparable results (Additional file 1: Table A2 and Table A3).

All analyses were performed with SPSS version 23.0 (IBM SPSS Statistics for Windows, Armonk, NY: IBM Corp). The level of significance was set at *P*-value< 0.05.

## Results

### Baseline characteristics of participants

The general characteristics of the study population at baseline are presented in Table 1. The participants’ average age was 79.8 (SD = 5.2) years old, with a range of 70 to 98 years, and 61.0% of them were female. Participants who engaged in moderated PA with a low frequency at baseline were older (*P < .001*), were more often female (*P < .001*), had less often completed tertiary level education (*P < .001*), were less often at risk for alcohol use (*P < .001*), more often suffered from multi-morbidity (*P* = 0.005), and were more often frail (*P < .001*) than those with a regular frequency at baseline.

**Table 1:** Baseline characteristics of participants in the analyses (*n* = 1614)

Characteristics	Total (n = 1614)	Baseline frequency of moderate physical activity			12-month change in physical activity				
		Regular frequency (*n* = 1171)	Low frequency (*n* = 443)	*P*-value	Continued regular frequency (*n* = 947)	Continued low frequency (*n* = 290)	Increased frequency (*n* = 153)	Decreased frequency (*n* = 224)	*P*-value
Age	79.8 ± 5.2	79.3 ± 4.9	81.2 ± 5.7	**<.001**^**a**^	79.0 ± 4.8	81.9 ± 5.8	80.0 ± 5.2	80.5 ± 5.2	**<.001**^**b**^
Gender									
*Female*	985 (61.0)	678 (57.9)	307 (69.3)	**<.001**^**c**^	543 (57.3)	211 (72.8)	96 (62.7)	135 (60.3)	**<.001**^**c**^
*Male*	629 (39.0)	493 (42.1)	136 (30.7)		404 (42.7)	79 (27.2)	57 (37.3)	89 (39.7)	
Country									
*Spain*	370 (22.9)	306 (26.1)	64 (14.4)	**<.001**^**c**^	270 (28.5)	24 (8.3)	40 (26.1)	36 (16.1)	**<.001**^**c**^
*Greece*	163 (10.1)	108 (9.2)	55 (12.4)		72 (7.6)	40 (13.8)	15 (9.8)	36 (16.1)	
*Croatia*	413 (25.6)	250 (21.3)	163 (36.8)		183 (19.3)	127 (43.8)	36 (23.5)	67 (29.9)	
*the Netherlands*	248 (15.4)	187 (16.0)	61 (13.8)		152 (16.1)	30 (10.3)	31 (20.3)	35 (15.6)	
*the United Kingdom*	420 (26.0)	320 (27.3)	100 (22.6)		270 (28.5)	69 (23.8)	31 (20.3)	50 (22.3)	
Educational level									
*Primary or less*	388 (24.2)	293 (25.2)	95 (21.5)	**<.001**^**c**^	239 (25.4)	57 (19.7)	38 (25.2)	54 (24.3)	**<.001**^**c**^
*Secondary*	1065 (66.4)	741 (63.7)	324 (73.5)		604 (64.1)	220 (75.9)	104 (68.9)	137 (61.7)	
*Tertiary*	152 (9.5)	130 (11.2)	22 (5.0)		99 (10.5)	13 (4.5)	9 (6.0)	31 (14.0)	
Living situation									
*Living alone*	622 (38.6)	437 (37.4)	185 (42.0)	0.093^c^	352 (37.2)	126 (43.4)	59 (39.1)	85 (38.1)	0.298^c^
*Living with others*	988 (61.4)	732 (62.6)	256 (58.0)		594 (62.8)	164 (56.6)	92 (60.9)	138 (61.9)	
Smoking									
*Yes*	118 (7.3)	94 (8.0)	24 (5.4)	0.075^c^	72 (7.6)	14 (4.9)	10 (6.5)	22 (9.8)	0.180^c^
*No*	1493 (92.7)	1076 (92.0)	417 (94.6)		874 (92.4)	274 (95.1)	143 (93.5)	202 (90.2)	
Alcohol risk									
*Yes*	410 (26.6)	339 (30.6)	71 (16.4)	**<.001**^**c**^	280 (31.1)	36 (12.7)	35 (23.6)	59 (28.4)	**<.001**^**c**^
*No*	1131 (73.4)	770 (69.4)	361 (83.6)		621 (68.9)	248 (87.3)	113 (76.4)	149 (71.6)	
Multi-morbidity									
*Yes*	1460 (90.5)	1045 (89.3)	415 (93.7)	**0.008**^**c**^	840 (88.7)	278 (95.9)	137 (89.5)	205 (91.9)	**0.003**^**c**^
*No*	153 (9.5)	125 (10.7)	28 (6.3)		107 (11.3)	12 (4.1)	16 (10.5)	18 (8.1)	
Frailty									
*Yes*	865 (53.8)	523 (44.8)	342 (77.6)	**<.001**^**c**^	393 (41.7)	240 (83.3)	102 (66.7)	130 (58.0)	**<.001**^**c**^
*No*	743 (46.2)	644 (55.2)	99 (22.4)		550 (58.3)	48 (16.7)	51 (33.3)	94 (42.0)	
Intervention condition									
*Yes (intervention group)*	864 (53.5)	649 (55.4)	215 (48.5)	**0.013**^**c**^	524 (55.3)	127 (43.8)	88 (57.5)	125 (55.8)	**0.003**^**c**^
*No (control group)*	750 (46.5)	522 (44.6)	228 (51.5)		423 (44.7)	163 (56.2)	65 (42.5)	99 (44.2)	

*Note*: Presented as mean ± SD or N(%)

Missing items: Education = 9; Live situation = 4; Smoke = 3; Alcohol = 73; Frailty = 6; Multi-morbidity = 1.

^a^ Independent T test, significant *P*-values in bold

^b^ One-way analysis of variance (ANOVA), significant *P*-values in bold

^c^ Chi-square tests, significant *P*-values in bold

**Fig. 1:**
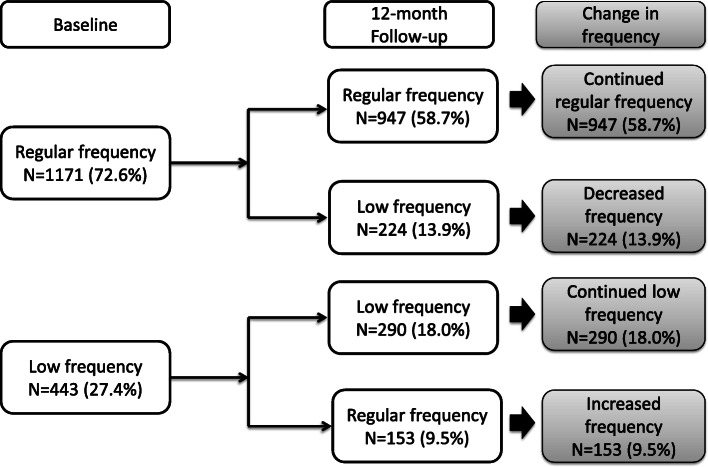
Frequency of moderate physical activity of participants

The frequency of moderate PA of participants at baseline and follow-up as well as change in frequency of moderate PA are presented in Fig. 1. At baseline, 1171 participants reported engaging in moderate PA with a regular frequency. After 12-month follow-up, 947 of these participants (58.7% of the study population) continued this regular frequency, while 224 of these participants (13.9%) had decreased to low frequency. At baseline, 443 participants reported engaging in moderate PA with a low frequency. Of these, 290 (18.0%) continued this low frequency after 12 months of follow-up, while 153 (9.5%) had increased to regular frequency.

### HRQoL at baseline and follow-up

**Fig. 2:**
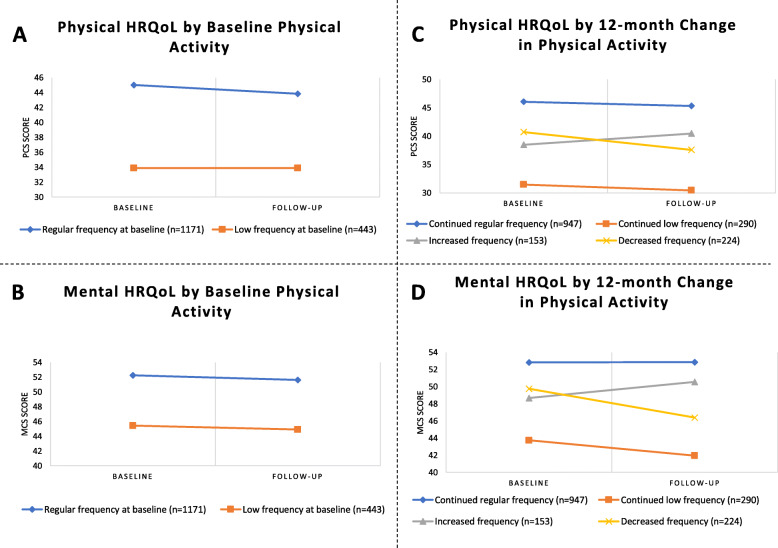
Health-related quality of life (HRQoL) at baseline and follow-up of participants from the groups of frequency of moderate physical activity: (**A**) Physical HRQoL by baseline physical activity, (**B**) Mental HRQoL by baseline physical activity, (**C**) Physical HRQoL by 12-month change in physical activity, and (**D**) Mental HRQoL by 12-month change in physical activity

The univariate analyses of physical and mental HRQoL at baseline and follow-up by 1) frequency of moderate PA at baseline, and 2) 12-month change in frequency of moderate PA are presented in Table 2 and Fig. 2.

**Table 2:** Baseline and follow-up mean scores of physical and mental health related quality of life of different physical activity groups

Frequency of PA	Mean score of physical HRQoL (PCS)			Mean score of mental HRQoL (MCS)		
	Baseline	Follow-up	*P*-value ^a^	Baseline	Follow-up	*P*-value ^a^
Baseline frequency of PA						
Regular frequency (*n* = 1171)	45.02 ± 10.33	43.86 ± 11.03	**<.001**^a^	52.23 ± 9.33	51.61 ± 10.17	**0.032**^**a**^
Low frequency (*n* = 443)	33.89 ± 12.12	33.89 ± 11.00	0.995^a^	45.44 ± 12.10	44.92 ± 12.91	0.309^a^
*P*-value ^b^	**<.001**^b^	**<.001**^b^		**<.001**^b^	**<.001**^b^	
*Effect size* ^*c, d*^	0.99	0.91		0.63	0.58	
Change in PA						
Continued regular frequency (n = 947)	46.04 ± 9.67	45.34 ± 10.47	**0.013**^a^	52.81 ± 8.98	52.85 ± 9.42	0.899^a^
Continued low frequency (n = 290)	31.48 ± 11.52	30.41 ± 9.56	**0.044**^a^	43.75 ± 12.32	41.95 ± 13.33	**0.003**^a^
Increased frequency (n = 153)	38.46 ± 11.95	40.48 ± 10.55	**0.011**^a^	48.66 ± 11.02	50.54 ± 9.91	**0.045**^a^
Decreased frequency (*n* = 224)	40.71 ± 11.81	37.58 ± 11.18	**<.001**^a^	49.75 ± 10.34	46.38 ± 11.52	**<.001**^a^
*P*-value ^e^	**<.001**^c^	**<.001**^c^		**<.001**^c^	**<.001**^c^	
*Effect size* ^*c, f*^	1.37	1.49		0.84	0.94	
*Effect size* ^*c, g*^	0.70	0.46		0.41	0.24	
*Effect size* ^*c, h*^	0.49	0.72		0.32	0.61	

*Note:* Presented as mean ± SD or N(%); Regular frequency = more than once a week; Low frequency = once a week or less; Increased frequency = from low to regular frequency; Decreased frequency = from regular to low frequency

Abbreviations: PA = physical activity; HRQoL = health-related quality of life; PCS = physical component summary; MCS = mental component summary

^a^ Paired T test of baseline and follow-up scores, significant *P*-values in bold

^b^ Independent T test of two Baseline frequency of PA groups, significant *P*-values in bold

^c^ Cohen’s effect sizes (d) were used for the interpretation of relevant differences: 0.20 ≤ d < 0.50 was considered a small difference; 0.50 ≤ d < 0.80 was considered a moderate difference; d ≥ 0.80 was considered a large difference

^d^ Cohen’s effect sizes (d) for differences in HRQoL between *Regular frequency of baseline physical activity* and *Low frequency of baseline physical activity* groups

^e^ One-way analysis of variance (ANOVA) of four Change in PA groups, significant *P*-values in bold

^f^ Cohen’s effect sizes (d) for differences in HRQoL between *Continued regular frequency of physical activity* and *Continued low frequency of physical activity* groups

^g^ Cohen’s effect sizes (d) for differences in HRQoL between *Continued regular frequency of physical activity* and *Increased frequency of physical activity* groups

^h^ Cohen’s effect sizes (d) for differences in HRQoL between *Continued regular frequency of physical activity* and *Decreased frequency of physical activity* groups

Participants who engaged in moderate PA with a low frequency had significantly poorer physical (*P < .001*; Fig. 2, Part A) and mental (*P < .001*; Fig. 2, Part B) HRQoL at follow-up than those who engaged in moderate PA with a regular frequency at baseline. A large difference in physical (d = 0.91) HRQoL and a moderate difference (d = 0.58) in mental HRQoL at follow-up were observed.

Part C and D of Fig. 2 show that participants who engaged in moderate PA with a continued regular frequency had the best physical and mental HRQoL among the four categories, while those with a continued low frequency had the poorest physical and mental HRQoL. Participants who decreased their frequency of moderate PA from regular to low had poorer physical (*P < .001*; Fig. 2, Part C) and mental (*P < .001*; Fig. 2, Part D) HRQoL at follow-up than at baseline. Conversely, participants who increased their frequency of moderate PA from low to regular had better physical (*P* = 0.011; Fig. 2, Part C) and mental (*P* = 0.045; Fig. 2, Part D) HRQoL at follow-up than at baseline.

Regarding physical HRQoL, a small difference (d = 0.49) at baseline and a moderate difference (d = 0.72) at follow-up between participants who engaged in moderate PA with a continued regular frequency and those who decreased their frequency of moderate PA from regular to low were observed. Conversely, a moderate difference (d = 0.70) at baseline and a small difference (d = 0.46) at follow-up between participants with a continued regular frequency and those who increased their frequency of moderate PA from low to regular were observed.

Regarding mental HRQoL, a small difference (d = 0.32) at baseline and a moderate difference (d = 0.61) at follow-up between participants who engaged in moderate PA with a continued regular frequency and those who decreased their frequency of moderate PA from regular to low were observed. While, small differences at baseline (d = 0.41) and follow-up (d = 0.24) between participants with a continued regular frequency and those who increased their frequency of moderate PA from low to regular were observed.

### Association between frequency of moderate PA and HRQoL

The associations between the baseline frequency of moderate PA and the follow-up physical and mental HRQoL according to the multivariate linear regression models are presented in Table 3. After adjusting for the HRQoL at baseline and all the covariates, participants who engaged in moderate PA with a low frequency at baseline had significantly poorer physical (B = -1.99; *P < .001*) and mental (B = -1.64; *P < .01*) HRQoL at follow-up than those with a regular frequency at baseline.

**Table 3:** Multivariate linear regression models (baseline frequency of physical activity and follow-up scores of physical and mental health related quality of life)

Baseline frequency of PA	12-month follow-up Physical HRQoL score		12-month follow-up Mental HRQoL score	
	Crude Model ^a^	Adjusted Model ^b^	Crude Model ^c^	Adjusted Model ^d^
*Regular frequency*	*Reference*	*Reference*	*Reference*	*Reference*
*Low frequency*	−2.46^***^	−1.99^***^	− 2.26^***^	− 1.64^**^
Adjusted R square, %	50.8	53.0	43.5	44.9

*Note:* Regular frequency = more than once a week; Low frequency = once a week or less; Increased frequency = from low to regular frequency; Decreased frequency = from regular to low frequency

Abbreviations: PA = physical activity; HRQoL = health-related quality of life

^a^ Crude Model: adjusted for country and baseline physical HRQoL score

^b^ Adjusted Model: adjusted for country, baseline physical HRQoL score as well as covariates including age, gender, education level, living situation, smoking, alcohol risk, multi-morbidity, frailty and intervention condition

^c^ Crude Model: adjusted for country and baseline mental HRQoL score

^d^ Adjusted Model: adjusted for country, baseline mental HRQoL score as well as covariates including age, gender, education level, living situation, smoking, alcohol risk, multi-morbidity, frailty and intervention condition

^*^p < .05, ^**^p < .01, ^***^p < .001, significant effect estimates in bold

### Association between 12-month change in frequency of moderate PA and HRQoL

The associations between 12-month change in frequency of moderate PA and physical and mental HRQoL at follow-up according to the multivariate linear regression models are presented in Table 4. A decrease in the frequency of moderate PA or a continued low frequency were significantly correlated with poorer physical and mental HRQoL at follow-up.

**Table 4:** Multivariate linear regression models (Change in physical activity and follow-up scores of physical and mental health-related quality of life)

Change in PA	12-month follow-up Physical HRQoL score		12-month follow-up Mental HRQoL score	
	Crude Model ^a^	Adjusted Model ^b^	Crude Model ^c^	Adjusted Model ^d^
*Continued regular frequency*	*Reference*	*Reference*	*Reference*	*Reference*
*Continued low frequency*	−6.01^***^	−5.45^***^	−4.75^***^	− 4.10^***^
*Increased frequency*	− 0.02	0.26	− 0.50	− 0.19
*Decreased frequency*	− 4.60^***^	−4.42^***^	− 4.15^***^	−3.95^***^
Adjusted R square, %	53.7	55.6	45.6	46.7

*Note:* Regular frequency = more than once a week; Low frequency = once a week or less; Increased frequency = from low to regular frequency; Decreased frequency = from regular to low frequency

Abbreviations: PA = physical activity; HRQoL = health-related quality of life

^a^ Crude Model: adjusted for country and baseline physical HRQoL score

^b^ Adjusted Model: adjusted for country, baseline physical HRQoL score as well as covariates including age, gender, education level, living situation, smoking, alcohol risk, multi-morbidity, frailty and intervention condition

^c^ Crude Model: adjusted for country and baseline mental HRQoL score

^d^ Adjusted Model: adjusted for country, baseline mental HRQoL score as well as covariates including age, gender, education level, living situation, smoking, alcohol risk, multi-morbidity, frailty and intervention condition

^*^p < .05, ^**^p < .01, ^***^p < .001, significant effect estimates in bold

After adjusting for physical HRQoL at baseline and all the covariates, participants who decreased their frequency of moderate PA from regular to low (B = -4.42; *P < .001*) and participants who continued the low frequency (B = -5.45; *P < .001*) had significantly poorer physical HRQoL at follow-up than those with a continued regular frequency. Similarly, after adjusting for mental HRQoL at baseline and all the covariates, participants who decreased their frequency of moderate PA from regular to low (B = -3.95; *P < .001*) and participants who continued the low frequency (B = -4.10; *P < .001*) had significantly poorer mental HRQoL than those with a continued regular frequency. Furthermore, there was no significant difference in physical and mental HRQoL at follow-up between participants who increased their frequency from low to regular and participants who continued a regular frequency.

## Discussion

The results of the present study showed that the frequency of moderate PA at baseline is positively associated with HRQoL at follow-up among community-dwelling older adults aged over 70; not only physical but also mental HRQoL. Furthermore, physical and mental HRQoL of participants who engaged in moderate PA with a continued regular frequency were relatively high at both baseline and follow-up. Participants who increased the frequency of moderate PA from low to regular had better physical and mental HRQoL at follow-up than themselves at baseline. After controlling for all covariates and baseline HRQoL, the follow-up physical and mental HRQoL of participants who increased their frequency from low to regular were similar to those who continued a regular frequency. These findings from both univariate analyses and multivariate linear regression models indicate that maintaining a regular frequency of PA as well as a pattern of increasing frequency of PA are associated with maintaining or improving physical and mental HRQoL among community-dwelling older adults aged over 70.

Some previous studies were in accordance with our findings. A 12-month cohort study conducted in Japan among older adults found that habitual PA, the daily step count and the daily duration of physical activity at an intensity > 3 metabolic equivalents (METs), was positively associated with both physical and mental domains of HRQoL [8]. An intervention study conducted in Iceland among older adults reported that a 12-week resistance exercise program significantly improved physical function and overall HRQoL [9]. A randomized controlled trial (RCT) conducted in the US among older adults found that PA interventions, walking plus strength and flexibility exercises, can slow the decline in overall HRQoL [27]. One potential explanation of these associations might be that PA may promote physical independence by improving functional capacity and physical health, which are positive changes in life [2]. These changes may have a positive influence on physical HRQoL. Moreover, some previous studies also observed positive effects of exercise on mental health and cognitive performance in adult populations, [28, 29] which might be a potential mechanism of the positive association between PA and mental HRQoL.

However, some studies reported different findings compared to our results. A 3-year cohort study conducted in France among adults reported no significant change in physical HRQoL with the change in leisure-time PA (h/week) [30]. A RCT conducted in Ghana among adults with type 2 diabetes reported no significant effects of an 8-week aerobic exercise training on overall HRQoL compared with usual care [11]. The differences might be caused by different study populations, study design and characteristics of PA intervention methods (e.g. the intensity, frequency, and duration of PA). More longitudinal and intervention studies are needed to investigate the optimum PA level to promote HRQoL for specific populations.

Finally, regarding mental HRQoL, we observed an interaction between the 12-month change in frequency of moderate PA and country; in the Netherlands the results were different from the results from the other four countries (see Additional file 1: Table A4). More studies are needed to clarify this finding.

### Strengths and limitations

The addition of empirical data regarding the relationship between change in PA and HRQoL among people aged over 70 years from a representative community-based survey in five European countries is a strength of our research. Furthermore, we applied the SF-12 to measure HRQoL, which has been validated in European countries including the UK, Greece, Croatia, The Netherlands and Spain, [31] to measure physical and mental HRQoL.

However, results of the current study should be interpreted in light of some limitations. First, PA was assessed using a single self-reported question that did not distinguish between types of activities or consider activity duration. We suggest future studies using a more rigorous measurement of PA, for example, the International Physical Activity Questionnaire, [32] to confirm our findings. Second, our analysis included participants in both the intervention and control groups. It’s possible that the intervention improved people’s health and lead to an overestimation of the impact of PA on HRQoL. However, we adjusted for the intervention condition by adding it as a covariate in the regression models. We also ran the analyses again for the control group only and found comparable results (See Additional file 1: Table A2 and Table A3). Third, participants who were relatively healthy may have participated in the research possibly causing selection bias. Fourth, our observational study cannot prove that PA and HRQoL are causally related. External causes such as an accident, stroke, fall, or other detrimental life circumstances during the year may also have contributed to a decline in PA. Adjusting for multi-morbidity and frailty at baseline only partially illustrates the changes in PA over the 12-month span. Additionally, there might be an overestimation of the association between changes in PA and HRQoL in the regression models since we only controlled for covariates at baseline. Further studies that adopted the study design of multiple repeated measurements, instead of baseline and follow-up only, are recommended to confirm our findings. Fifth, overadjustment bias could arise since we controlled for a large number of covariates, some of which (such as multimorbidity and frailty) may serve both as a confounder and a mediator. On the other hand, although we tried to capture the most important confounding factors, there could still be residual confounding. Sixth, there may be some overlap between PA and two items in physical functioning domain of the SF-12, which might lead to an overestimation of the relationship. However, when we investigated the relationship between PA and physical HRQoL, the results were comparable after excluding these two items. As a result, we do not anticipate that this limitation has changed our findings. Seventh, there is an interaction effect between country and change in moderate PA on mental HRQoL (Additional file 1: Table A1). We recommend future studies to explore this association and to clarify these findings.

## Conclusions

In conclusion, our findings indicated that both maintaining a regular frequency of PA and increasing activity during a year to a regular frequency of PA are associated with maintaining or improving physical and mental HRQoL among European community-dwelling older adults. Our findings support the development of health promotion and long-term care strategies to encourage older adults to maintain regular frequency of PA to promote their quality of life. More longitudinal and intervention studies on the effect of the frequency and intensity levels of PA on HRQoL are needed to better understand and determine the optimum level of PA to promote HRQoL among older adults.

## Supplementary Information

**Additional file 1.**

## Data Availability

The datasets used and/or analysed during the current study are available from the corresponding author on reasonable request.

## Abbreviations

HRQoL: Health-related quality of life
ISCED: International Standard Classification of Education
MCS: Mental Component Summary
PA: Physical activity
PCS: Physical Component Summary
RCT: randomized controlled trial
SF-12: 12-Item Short-Form Health Survey
SHARE-FI: Frailty Instrument of the Survey of Health, Aging and Retirement in Europe
TFI: Tilburg Frailty Indicator
UHCE: Urban Health Centres Europe
