# Supplementary material for: Longitudinal association between physical activity and health-related quality of life among community-dwelling older adults: a longitudinal study of Urban Health Centres Europe (UHCE)
Source: BMC Geriatr. 2021 Oct 1;21:521. doi: 10.1186/s12877-021-02452-y (PMC8485559; doi:10.1186/s12877-021-02452-y)
Supplement: Supplementary file 1 — Additional file 1. [file 12877_2021_2452_MOESM1_ESM.docx]

**Additional file legends**

Additional file 1: Table A1 P-values for interactions between baseline frequency of moderate PA or 12-month change in frequency of moderate PA and age, sex, county, educational level, living situation and intervention on the health-related quality of life scores

Additional file 1: Table A2 Sensitivity analysis: multivariate linear regression models (baseline frequency of physical activity and follow-up scores of physical and mental health-related quality of life; control group only; n=750)

Additional file 1: Table A3 Sensitivity analysis: multivariate linear regression models (change in physical activity and follow-up scores of physical and mental health-related quality of life; control group only; n=750)

Additional file 1: Table A4 Multivariate linear regression models (change in physical activity and follow-up scores of mental health-related quality of life) stratified by country

**Additional file 1: Table A1** *P*-values for interactions between baseline frequency of moderate PA or 12-month change in frequency of moderate PA and age, sex, county, educational level, living situation and intervention on the health-related quality of life scores

| Items | 12-month follow-up physical HRQoL score | 12-month follow-up mental HRQoL score |
| --- | --- | --- |
|  | *P*-value | *P*-value |
| *Interactions in models on associations between baseline frequency of moderate PA and HRQoL at follow-up* |  |  |
| Baseline frequency of moderate PA*Age | 0.366 | 0.714 |
| Baseline frequency of moderate PA*Sex | 0.751 | 0.190 |
| Baseline frequency of moderate PA*Country | 0.163 | 0.241 |
| Baseline frequency of moderate PA*Educational level | 0.626 | 0.627 |
| Baseline frequency of moderate PA*Live situation | 0.505 | 0.559 |
| Baseline frequency of moderate PA*Intervention | 0.836 | 0.837 |
|  |  |  |
| *Interactions in models on associations between 12-month change in moderate PA and HRQoL at follow-up* |  |  |
| Change in moderate PA*Age | 0.327 | 0.129 |
| Change in moderate PA*Sex | 0.581 | 0.325 |
| Change in moderate PA*Country | 0.181 | **<.001** |
| Change in moderate PA*Educational level | 0.800 | 0.792 |
| Change in moderate PA*Live situation | 0.130 | 0.486 |
| Change in moderate PA*Intervention | 0.477 | 0.550 |
| *Note*: After applying Bonferroni correction for multiple testing (P=0.05/12=0.004), except interaction between country and change in physical activity on follow-up mental HRQoL score, no statistically significant interaction was found.  Abbreviations: PA = physical activity; HRQoL = health-related quality of life.  Significant *P*-values in bold. | | |

**Additional file 1: Table A2** Sensitivity analysis: multivariate linear regression models (baseline frequency of physical activity and follow-up scores of physical and mental health-related quality of life; control group only; n=750)

| Baseline frequency of PA | 12-month follow-up  Physical HRQoL score | | 12-month follow-up  Mental HRQoL score | |
| --- | --- | --- | --- | --- |
|  | Crude Model ^a^ | Adjusted Model ^b^ | Crude Model ^c^ | Adjusted Model ^d^ |
| *Regular frequency* | *Reference* | *Reference* | *Reference* | *Reference* |
| *Low frequency* | **-2.62^***^** | **-2.45^**^** | **-2.09^**^** | -1.43 |
| Adjusted R square, % | 54.4 | 56.4 | 47.4 | 49.2 |
| *Note:* Abbreviations: PA = physical activity; HRQoL = health-related quality of life.  ^a^ Crude Model: adjusted for country and baseline physical HRQoL score.  ^b^ Adjusted Model: adjusted for country, baseline physical HRQoL score as well as covariates including age, gender, education level, living situation, smoking, alcohol risk, multi-morbidity and frailty.  ^c^ Crude Model: adjusted for country and baseline mental HRQoL score.  ^d^ Adjusted Model: adjusted for country, baseline mental HRQoL score as well as covariates including age, gender, education level, living situation, smoking, alcohol risk, multi-morbidity and frailty.  ^*^*p*<.05, ^**^*p*<.01, ^***^*p*<.001, significant effect estimates in bold. | | | | |

**Additional file 1: Table A3** Sensitivity analysis: multivariate linear regression models (change in physical activity and follow-up scores of physical and mental health-related quality of life; control group only; n=750)

| Change in PA | 12-month follow-up  Physical HRQoL score | | 12-month follow-up  Mental HRQoL score | |
| --- | --- | --- | --- | --- |
|  | Crude Model ^a^ | Adjusted Model ^b^ | Crude Model ^c^ | Adjusted Model ^d^ |
| *Continued regular frequency* | *Reference* | *Reference* | *Reference* | *Reference* |
| *Continued low frequency* | **-5.30^***^** | **-4.97^***^** | **-4.35^***^** | **-3.60^***^** |
| *Increased frequency* | -0.20 | -0.08 | 0.66 | 0.90 |
| *Decreased frequency* | **-4.19^***^** | **-3.38^***^** | **-3.64^***^** | **-3.36^**^** |
| Adjusted R square, % | 56.4 | 58.1 | 49.5 | 50.8 |
| *Note:* Abbreviations: PA = physical activity; HRQoL = health-related quality of life.  ^a^ Crude Model: adjusted for country and baseline physical HRQoL score.  ^b^ Adjusted Model: adjusted for country, baseline physical HRQoL score as well as covariates including age, gender, education level, living situation, smoking, alcohol risk, multi-morbidity, frailty and intervention.  ^c^ Crude Model: adjusted for country and baseline mental HRQoL score.  ^d^ Adjusted Model: adjusted for country, baseline mental HRQoL score as well as covariates including age, gender, education level, living situation, smoking, alcohol risk, multi-morbidity, frailty and intervention.  ^*^*p*<.05, ^**^*p*<.01, ^***^*p*<.001, significant effect estimates in bold. | | | | |

**Additional file 1: Table A4** Multivariate linear regression models (change in physical activity and follow-up scores of mental health-related quality of life) stratified by country

| Change in PA | Spain | | Greece | | Croatia | | The Netherlands | | The United Kingdom | |
| --- | --- | --- | --- | --- | --- | --- | --- | --- | --- | --- |
|  | 12-month follow-up  Mental HRQoL score | | 12-month follow-up  Mental HRQoL score | | 12-month follow-up  Mental HRQoL score | | 12-month follow-up  Mental HRQoL score | | 12-month follow-up  Mental HRQoL score | |
|  | Crude Model ^a^ | Adjusted Model ^b^ | Crude Model ^a^ | Adjusted Model ^b^ | Crude Model ^a^ | Adjusted Model ^b^ | Crude Model ^a^ | Adjusted Model ^b^ | Crude Model ^a^ | Adjusted Model ^b^ |
| *Continued regular frequency* | *Reference* | *Reference* | *Reference* | *Reference* | *Reference* | *Reference* | *Reference* | *Reference* | *Reference* | *Reference* |
| *Continued low frequency* | **-6.26 ^**^** | **-6.07^**^** | **-5.84^***^** | **-5.78^**^** | **-7.33^***^** | **-6.84^***^** | 1.49 | **3.28^*^** | **-3.41^**^** | **-0.3.07^**^** |
| *Increased frequency* | -0.67 | -0.90 | -1.83 | -1.30 | 0.54 | 0.50 | -3.18 | -2.37 | 1.48 | 1.84 |
| *Decreased frequency* | **-9.58^***^** | **-9.46^***^** | **-2.12^*^** | **-1.68*** | **-6.90^***^** | **-6.88^***^** | 0.58 | 1.38 | -1.71 | -1.77 |
| Adjusted R square, % | 34.9 | 36.1 | 41.5 | 41.7 | 40.7 | 42.4 | 19.2 | 23.7 | 22.2 | 22.8 |
| *Note:* Abbreviations: PA = physical activity; HRQoL = health-related quality of life.  ^a^ Crude Model: adjusted for baseline mental HRQoL score.  ^b^ Adjusted Model: adjusted forbaseline mental HRQoL score and covariates including age, gender, education level, living situation, smoking, alcohol risk, multi-morbidity, frailty and intervention.  ^*^*p*<.05, ^**^*p*<.01, ^***^*p*<.001, significant effect estimates in bold. | | | | | | | | | | |
